# A respiratory/Hirschsprung phenotype in a three‐generation family associated with a novel pathogenic *PHOX2B* splice donor mutation

**DOI:** 10.1002/mgg3.1528

**Published:** 2020-10-13

**Authors:** Nikolai Paul Pace, Michael Pace Bardon, Isabella Borg

**Affiliations:** ^1^ Centre for Molecular Medicine and Biobanking Faculty of Medicine and Surgery University of Malta Msida Malta; ^2^ Department of Medicine Mater Dei Hospital Msida Malta; ^3^ Medical Genetics Unit Department of Pathology Mater Dei Hospital Msida Malta; ^4^ Department of Pathology Faculty of Medicine and Surgery University of Malta Msida Malta

**Keywords:** Congenital Central Hypoventilation Syndrome, Hirschsprung disease, *PHOX2B* gene

## Abstract

**Background:**

Mutations in the *PHOX2B* gene cause congenital central hypoventilation syndrome (CCHS), a rare autonomic nervous system dysfunction disorder characterized by a decreased ventilatory response to hypercapnia. Affected subjects develop alveolar hypoventilation requiring ventilatory support particularly during the non‐REM phase of sleep. In more severe cases, hypoventilation may extend into wakefulness. CCHS is associated with disorders characterized by the defective migration/differentiation of neural crest derivatives, including aganglionic megacolon or milder gastrointestinal phenotypes, such as constipation. Most cases of CCHS are de novo, caused by heterozygosity for polyalanine repeat expansion mutations (PARMs) in exon 3. About 10% of cases are due to heterozygous non‐PARM missense, nonsense or frameshift mutations.

**Methods:**

We describe a three‐generation Maltese‐Caucasian family with a variable respiratory/Hirschsprung phenotype, characterized by chronic constipation, three siblings with Hirschsprung disease necessitating surgery, chronic hypoxia, and alveolar hypoventilation requiring non‐invasive ventilation.

**Results:**

The sequencing of *PHOX2B* revealed a novel heterozygous c.241+2delT splice variant in exon 1 that segregates with the CCHS/Hirschsprung phenotype in the family. The mutation generates a non‐functional splice site with a deleterious effect on protein structure and is pathogenic according to ACMG P VS1, PM2, and PP1 criteria.

**Conclusion:**

This report is significant as no *PHOX2B* splice‐site mutations have been reported. Additionally, it highlights the variability in clinical expression and disease severity of non‐PARM mutations.

## Graphical abstract

Mutations in the PHOX2B gene cause congenital central hypoventilation syndrome (CCHS), a rare autonomic nervous system dysfunction disorder characterized by a decreased ventilatory response to hypercapnia. We describe a three‐generation family with a variable respiratory/Hirschsprung phenotype caused by a novel c.241+2delT splice donor mutation in exon 1. This report is significant as no PHOX2B splice‐site mutations have been reported to date.

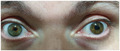

## 1 INTRODUCTION

Mutations in the paired like homeobox 2B gene (*PHOX2B* NM_003924.4, HGNC:9143) cause congenital central hypoventilation syndrome (CCHS—OMIM #209880). This rare disorder is characterized by autonomic dysfunction, leading to a reduced ventilatory response to hypercapnia and hypoxia in both wakefulness and sleep. (Mellins et al., 1970; Paton et al., 1989). Affected subjects develop sustained hypoxia and hypercapnia in the absence of cardiac, pulmonary or neuromuscular abnormalities that requires ventilatory support (Weese‐Mayer et al., 2010). CCHS generally presents with apnea or hypercapnia in the neonatal period, although later‐onset presentations in the postneonatal period or adulthood have been described (Antic et al., 2006; Bittencourt et al., 2012). The clinical severity of the respiratory phenotype in CCHS is highly variable and may be associated with lower‐penetrance disorders caused by the defective migration or differentiation of neural crest derivatives such as aganglionic megacolon or tumors of neural crest origin.

*PHOX2B* encodes a homeobox transcription factor that controls the development of reflex circuits in the autonomic nervous system. Most cases (90%) are heterozygotes for in‐frame triplet duplications within exon 3 in the region coding for the 20 Ala residues, leading to the expansion of the polyAla stretch (termed PARMs, polyAla repeat expansion mutations). About 10% of cases are due to heterozygous missense, nonsense or frameshift mutations in the coding exons (non‐PARM mutations) (Amiel et al., 2003; Trochet et al., 2005; Weese‐Mayer et al., 2003). Non‐PARMs are commonly associated with syndromic phenotypes; however, a wide variation in phenotypic expressivity of non‐PARMs is reported. A subset of mutations has been associated with milder phenotypes, such as isolated Hirschsprung disease or neuroblastomas (Garcia‐Barceló et al., 2003; Mosse et al., 2004). We describe a variable CCHS/Hirschsprung phenotype in a three‐generation family arising from a novel disruption in the *PHOX2B* exon 1 canonical donor splice site.

## 2 PATIENT PHENOTYPES

This study was approved by the local institutional ethics review board and written informed consent for genetic studies was obtained.

The proband was a female infant born to non‐consanguineous parents. The mother is of Maltese‐Caucasian ethnicity. She was delivered at 35 weeks gestation by emergency Caesarean section in view of fetal distress after a pregnancy complicated by gestational hypertension and intrauterine growth retardation. Dysmorphic features suggestive of Trisomy 21 were noted at birth, and G‐banded chromosome analysis revealed a 47,XX+21 karyotype. Cardiac echocardiography was normal.

The proband's Apgar score was 9 at one and five minutes postnatally. Within the first 24 h, she developed frequent bradypnea and apnoeic episodes leading to type 2 respiratory failure, with a resting HCO_3_ level of 33 mmol/L and a PCO_2_ of 10.1 kPa, thus requiring admission to neonatal intensive care and initiation of Bi‐level Positive Pressure Ventilation (BiPAP). No obvious etiology underlying central hypoventilation was present. Additionally, no meconium was passed in the first 24 h, and she subsequently developed abdominal distension and bilious vomiting. Histopathological analysis of colonic biopsies on day 2 demonstrated absent submucosal ganglion cells and hypertrophic nerve trunks, consistent with aganglionic megacolon. The proband underwent colonic resection and defunctioning ileostomy two weeks postnatally, and could not be weaned off invasive mechanical ventilatory support in view of worsening respiratory failure in the postoperative period. She subsequently developed recurrent severe lower respiratory tract infections and passed away at five months of age.

The proband's mother (aged 46) suffers from multifactorial chronic respiratory failure with a significant impairment of exercise tolerance (Modified MRC Dyspnea scale 4, WHO Performance status 2). Domiciliary nocturnal BiPAP as well as continuous oxygen therapy were initiated at age 41 when the diagnosis of CCHS had already been established during family segregation analysis of the pathogenic mutation identified in the neonate. Her clinical deterioration was reflected by the progressive worsening of hypoxemia and hypercapnia (resting PCO_2_ level of 10 kPa aged 46 compared to 7.2 kPa aged 43). Notable findings on clinical examination include central cyanosis and anisocoria with a left‐sided D‐shaped pupil that reacts slowly to light and accommodation. The exertional dyspnea is also exacerbated by underlying interstitial lung disease associated with positive avian precipitins, requiring steroid therapy. High‐resolution pulmonary CT revealed changes reminiscent of pulmonary hypertension secondary to chronic hypoxia, and trans‐thoracic echocardiography revealed the presence of a dilated and hypo‐contractile right ventricle. She also suffers from chronic constipation. Since she refused investigation for intestinal pathology a formal diagnosis of Hirschsprung disease could not be made.

The proband's maternal grandmother has a longstanding history of chronic constipation and mild exertional dyspnea; however, she rarely seeks or requires medical attention. She has never developed respiratory failure, nor has she ever required non‐invasive ventilation. She shares the same D‐shaped pupil anomaly as her daughter.

**FIGURE 1:**
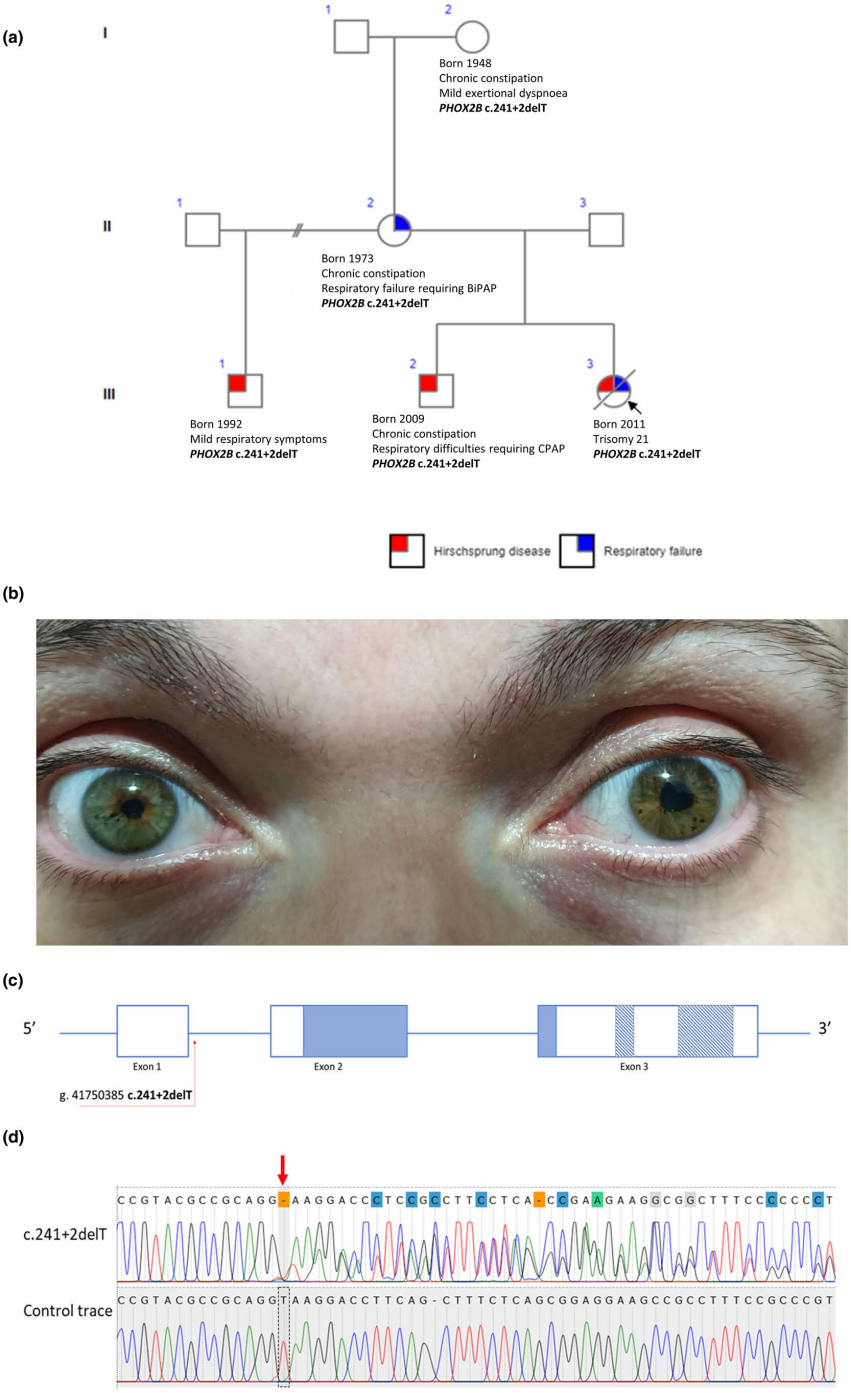
(a) Pedigree describing salient clinical features in the kindred. The proband (III.3) presented with features of trisomy 21, Hirschsprung disease, and chronic respiratory failure requiring invasive ventilation. Her brothers (III.1 and III.2) exhibit varying severity of the respiratory failure. Subject II.2 has a chronic hypoxemic hypercapnic respiratory failure with severe limitation of exercise tolerance requiring BiPAP. Her respiratory failure is multifactorial in etiology. (b) D‐Shaped pupil in subject II.2. (c) Diagrammatic representation of the *PHOX2B* NM_003924.4 gene, showing the location of the mutation identified in the family described in this report. Solid blue areas in exons 2 and 3 represent the DNA‐binding homeobox domain. The thatched areas represent the polyalanine repeat stretches in exon 3. (d) Sanger sequencing trace of the *PHOX2B* exon 1‐intron 1 boundary, showing the heterozygous c.241+2delT deletion (red arrow) leading to the disruption of the GT splice donor site. Downstream of the deletion, the superimposed traces caused by chromatogram shift due to the heterozygous deletion can be observed. A comparison control trace is shown in the alignment.

The proband has two male siblings. The elder sibling is the half‐brother (III.1), who was diagnosed with histologically proven Hirschsprung disease and underwent Swenson's proctosigmoidectomy and pull‐through at three months of age. He subsequently developed mild respiratory symptoms, although the significance of these symptoms remains unknown as he was lost to clinical follow‐up. Her brother (III.2) similarly developed signs of intestinal obstruction with vomiting in the first few days of life. Colonic biopsies revealed aganglionic rectal and sigmoid colon, and a colostomy was performed at 3 weeks, followed by a Swenson's pull‐through procedure at 6 months. He subsequently developed recurrent episodes of dyspnea associated with nocturnal desaturation. Following the diagnosis of CCHS at the age of 2 years, polysomnography with overnight capnometry revealed nocturnal hypercapnia, (mean overnight PCO_2_ = 6.5 kPa maximum PCO_2_ = 8.1 kPA) as well as nocturnal hypoxemia with a minimum oxygen saturation of 79%. He was, therefore, commenced on domiciliary nocturnal CPAP. The patient remains poorly compliant to therapy, and reassessment by capnometry was never performed. A pedigree summarizing key clinical features in this kindred is shown in Figure 1a,b.

## 3 GENETIC ANALYSIS

In view of the clinical presentation and the unexplained respiratory failure requiring mechanical ventilation in the proband, CCHS was suspected. Screening for *PHOX2B* (NM_003924.4) mutations and extended family segregation studies was thus undertaken. A detailed description of the methods is provided in the supplementary file.

In all family members analyzed (I.2, II.2, III.1, III.2, III.3), fragment analysis revealed no polyalanine repeat expansion mutation, with homozygosity for the normal sized 20‐alanine tract detected. No missense, nonsense or frameshift variants within the protein‐coding regions were identified. A deletion in the invariant consensus splice donor site within intron 1 was detected in the proband (III.3) that segregates with the CCHS/Hirschsprung phenotype within the family (Figure 1c,d). The deletion—NM_003924.3:c.241+2delT, position 41750385 in hg19 reference genome assembly—is novel and is not reported in the Human Gene Mutation Database®, NCBI ClinVar, and LOVD databases. The splice donor site deletion meets the following ACMG/AMP criteria for pathogenicity (1) PVS1‐ pathogenic, very strong—a null variant occurring within ±2 bases of a splice site affecting gene *PHOX2B*, where the loss of function is a known mechanism of disease; (2) PM2—Pathogenic, Moderate—Variant not found in GnomAD exomes and GnomAD genomes despite good coverage and (3) PP3—pathogenic supporting—high evolutionary conservation scores (GERP = 5.5599) with no benign predictions. Additionally, the segregation of the mutation in a gene known to cause the disease in multiple affected family members provides additional support for pathogenicity (PP1 criterion). The c.241+2delT deletion leads to the disruption of the canonical wild‐type splice donor site, with alteration of the splicing mechanism through either exon skipping or use of cryptic sites. In silico analysis using different splice site prediction tools was implemented (Table 1). The predictions from both Human Splicing Finder (HSF) and MaxEntScan (MES) are deleterious according to Houdayer's criteria (Houdayer, 2011; Houdayer et al., 2012). Both algorithms calculate a decrease of more than 5% and 15%, respectively, from the wild‐type scores using SSF‐like and MES. Both NetGene2 and NNSplice indicated that the mutated sequence is not likely to be a functional splice donor site. When considered in combination, these scores indicate a high likelihood of splicing modification with a resulting deleterious impact on the protein sequence.

**TABLE 1:** Results from in silico analysis of the splice donor site of the intron 1. Scores for wild‐type and the mutant c.241_2delT sequence are presented. A sequence is predicted to be a functional splice site if the score is higher than the given threshold value

In silico splice analysis tool	5’ Motif score—Wild type	5’ Motif score—c.241_2delT	% variation	Threshold
Human splicing finder	98.07	62.49	−36.28	65
MaxEntScan	11.08	1.6	−85.56	3
NNSplice	1	Splice site not recognized		0.4
NetGene2	0.93	Splice site not recognized		0.5

## 4 DISCUSSION

We describe a novel *PHOX2B* splice donor site mutation that segregates with a complex CCHS‐Hirschsprung phenotype in a three‐generation family. The non‐PARM mutation described here exhibits an autosomal dominant pattern of inheritance, variable penetrance, and marked phenotypic heterogeneity. The highly variable nature of clinical phenotypes associated with *PHOX2B* non‐PARMs has been reported in the literature (Low et al., 2014). Most non‐PARMs have been reported to occur de novo and in the context of a more severe syndromic phenotype. However, the multigenerational transmission of non‐PARMs with milder presentations in late childhood or adulthood have been reported (Kasi et al., 2017). This is consistent with the clinical picture outlined in this report. The phenotypic variability in CCHS has been ascribed to multiple factors, including incomplete penetrance. Additionally, a range of somatic mosaicism has been reported in parents of children with CCHS, and germline mosaicism has also been demonstrated (Rand et al., 2012). The effect of modifier genes, such as *RET* in Hirschsprung disease as well as the possible role of environmental factors have been proposed to explain clinical heterogeneity (Amin et al., 2011).

A variety of non‐PARMs in the coding region of the gene have been described and are recently reviewed (Bachetti & Ceccherini, 2020). This report represents the first description of a splice donor site pathogenic variant in *PHOX2B*. However, Berry‐Kravis *et al* describe two recurrent missense substitutions in exon 2 (p.Ala428Gly and p.Gly422Ala) that are both predicted to alter residues in the splice donor consensus sequence essential for the splicing of exons 2 and 3 that can exert an effect through a splicing defect (Berry‐Kravis et al., 2006). Similarly, the rare synonymous substitution in exon 1 (*PHOX2B* c.270C>T, p.Gly90=) is predicted to alter the splicing mechanism and has a variant of uncertain significance entry in ClinVar with conflicting interpretations of pathogenicity.

The *PHOX2B* c.241+2delT mutation meets the criteria for pathogenicity according to ACMG consensus guidelines and this is further supported by splice site prediction algorithms. Substitutions that alter conserved canonical acceptor or donor sites that define exon‐intron boundaries impair the activity of the spliceosome, and this is a recognized pathogenic mechanism in multiple monogenic diseases (Fang et al., 2001). Canonical splice site substitutions can lead to exon skipping. Alternatively, if the native splice site is weak, a mutation can uncover a cryptic splice site in an adjacent exon or intron, leading to intronic fragment inclusion or exclusion of exonic fragments from mRNA isoforms (Anna & Monika, 2018). It must be emphasized that analysis through minigene assays and/or RNA sequencing is necessary to identify nonsense‐mediated decay (NMD) and characterize the effect of splice site mutations on transcript abundance or the activation of cryptic splice sites.

Most non‐PARMs are frameshifts located in exon 3 of *PHOX2B*. Of particular interest in this report are the functional similarities that can be drawn from comparison with cases bearing nonsense mutations in exon 1. Few such cases have been published (c.18 T > C p.Tyr6X, c.23delA p.Tyr8X, c.42C > A p.Tyr14X, c.13G > T, p.Glu5X (Cain et al., 2017; Magalhães et al., 2015; Parodi et al., 2008; Trochet et al., 2009). These exon 1 truncating mutations are characterized by relatively mild phenotypes, although there is a variation with regards to the age of onset of respiratory failure. Interestingly, no aganglionic megacolon was reported in these cases. Cain et al. showed that exon 1 nonsense mutations do not result in *PHOX2B* haploinsufficiency through nonsense‐mediated transcript decay. Rather, translational re‐initiation using an alternative start codon (Met 18 or Met 21) results in N‐terminally truncated proteins that retain functionality, and the truncated proteins localize to the nucleus and retain the capacity to transactivate target promoters (Cain et al., 2017). We hypothesize that the *PHOX2B* c.241+2delT mutation results in the generation of a transcript through the activation of a cryptic splice site which escapes NMD and forms a protein with hypomorphic properties analogous to that observed in exon 1 nonsense mutations.

In conclusion, the pathogenic splice donor site mutation in exon 1 of *PHOX2B* described here expands the molecular spectrum associated with this complex syndrome. A clear association between trisomy 21 and Hirschsprung disease exists, with a 40‐fold greater risk of Hirschsprung disease in neonates with Down Syndrome (Arnold et al., 2009). The coexistence of the two conditions leads to higher complication rates, poor functional outcomes, and increased mortality (Friedmacher & Puri, 2013). Down syndrome is also associated with hypoventilation, possibly due to respiratory muscle hypotonia and altered ventilatory control (Fan et al., 2017; Richard et al., 2020). The proband in this report bears a striking similarity to that described by Jones et al, where Hirschsprung disease and trisomy 21 were reported in a male neonate bearing a *PHOX2B* PARM (Jones et al., 2012). Both cases were characterized by failure to achieve successful extubation and the coexisting aneuploidy likely explains the severe presentation. Facial dysmorphology has also been described in children with CCHS, with a shorter flatter facies and a decreased upper facial height resulting in boxlike features (Todd et al., 2006). In keeping with Jones et al, the typical trisomy 21 phenotype was evident at birth in the proband, and this may have obscured facial features of CCHS. In the proband's mother, the severe limitation of exercise tolerance is compounded by the underlying interstitial lung disease. Although it could not be confirmed by histological examination, it is highly plausible that her gastrointestinal symptoms are attributable to mild Hirschsprung disease given the presence of anisocoria, respiratory insufficiency, and a pathogenic *PHOX2B* variant. Of note, none of the mutation carriers in this kindred had evidence of neural crest tumors, autonomic dysfunction on routine electrocardiography, and dysmorphism.

This report also emphasizes that a non‐PARM can escape clinical detection as it does not necessarily present with severe respiratory compromise in the neonatal period. As genetic testing becomes more widely accessible, it is likely that non‐PARMs with milder respiratory symptoms are recognized. The *PHOX2B* c.241+2delT mutation thus warrants further functional evaluation as the severity of clinical features in CCHS depends partly on the precise nature and location of the mutation.

## CONFLICT OF INTEREST

The authors declare that they have no conflict of interest.

## AUTHOR CONTRIBUTIONS

All authors contributed equally to the design and execution of this study, and reviewed and approved the manuscript for final publication.

## Supporting information

Supplementary Material
