# Supplementary material for: A respiratory/Hirschsprung phenotype in a three‐generation family associated with a novel pathogenic PHOX2B splice donor mutation
Source: Mol Genet Genomic Med. 2020 Oct 13;8(12):e1528. doi: 10.1002/mgg3.1528 (PMC7767558; doi:10.1002/mgg3.1528)
Supplement: Supplementary file 1 — Supplementary Material [file MGG3-8-e1528-s001.docx]

**Supplementary methods**

**Title: A respiratory/Hirschsprung phenotype in a three-generation family associated with a novel pathogenic *PHOX2B* splice donor mutation.**

**Methodology**

Genomic DNA was extracted from peripheral blood samples taken from the probands using a QIAamp DNA extraction kit (Qiagen, Hilden, Germany), and checked for purity and integrity using agarose gel electrophoresis and UV spectrophotometry. PCR amplification, fragment analysis and bi-directional Sanger sequencing of *PHOX2B* amplicons was carried out as follows. PCR primer sequences and cycling conditions were obtained from the literature (Garcia-Barceló et al., 2003). The three coding regions of *PHOX2B* (reference sequence NM_003924.3) and their flanking 5’ and 3’ sequences were amplified in a 25µL reaction volume containing 40ng of DNA, 0.5uM of each primer and 2x PCRBIO UltraMix Red (PCR Biosystems, UK) for amplification of GC-rich regions according to the manufacturers’ instructions. PCR products underwent spin column purification with a MinElute Reaction Cleanup Kit (Qiagen) and bi-directional Sanger sequencing using a dye terminator cycle sequencing kit (ABI PRISM Big Dye Terminator v 2.0) and an ABI 3100 sequencer (Applied Biosystems). The detected variants were analysed for pathogenicity according to established guidelines from the American College of Medical Genetics/Association for Molecular Pathology (ACMG/AMP) (Richards et al., 2015). These guidelines standardise the process of variant classification by stratification into five categories (pathogenic, likely pathogenic, uncertain significance, likely benign, benign) based on a combination of computational, population, functional and segregation data. The strength of splice site variants was evaluated using MaxEntScan, Human Splicing Finder, NNSplice and NetGene2. (Brunak, Engelbrecht, & Knudsen, 1991; Desmet et al., 2009; Reese, Eeckman, Kulp, & Haussler, 1997; Yeo & Burge, 2004).

**References**

Brunak, S., Engelbrecht, J., & Knudsen, S. (1991). Prediction of human mRNA donor and acceptor sites from the DNA sequence. *Journal of Molecular Biology*, *220*(1), 49–65. doi: 10.1016/0022-2836(91)90380-o

Desmet, F.-O., Hamroun, D., Lalande, M., Collod-Béroud, G., Claustres, M., & Béroud, C. (2009). Human Splicing Finder: An online bioinformatics tool to predict splicing signals. *Nucleic Acids Research*, *37*(9), e67. doi: 10.1093/nar/gkp215

Garcia-Barceló, M., Sham, M. H., Lui, V. C. H., Chen, B. L. S., Ott, J., & Tam, P. K. H. (2003). Association study of PHOX2B as a candidate gene for Hirschsprung’s disease. *Gut*, *52*(4), 563–567.

Reese, M. G., Eeckman, F. H., Kulp, D., & Haussler, D. (1997). Improved splice site detection in Genie. *Journal of Computational Biology: A Journal of Computational Molecular Cell Biology*, *4*(3), 311–323. doi: 10.1089/cmb.1997.4.311

Richards, S., Aziz, N., Bale, S., Bick, D., Das, S., Gastier-Foster, J., … ACMG Laboratory Quality Assurance Committee. (2015). Standards and guidelines for the interpretation of sequence variants: A joint consensus recommendation of the American College of Medical Genetics and Genomics and the Association for Molecular Pathology. *Genetics in Medicine: Official Journal of the American College of Medical Genetics*, *17*(5), 405–424. doi: 10.1038/gim.2015.30

Yeo, G., & Burge, C. B. (2004). Maximum entropy modeling of short sequence motifs with applications to RNA splicing signals. *Journal of Computational Biology: A Journal of Computational Molecular Cell Biology*, *11*(2–3), 377–394. doi: 10.1089/1066527041410418
